# *PLOS Genetics* 2016 Reviewer and Editorial Board Thank You

**DOI:** 10.1371/journal.pgen.1006671

**Published:** 2017-03-20

PLOS and the *PLOS Genetics* team would like to express our sincere gratitude to everyone who lent us their expertise as an associate editor, as a guest editor, or as a peer reviewer in 2016. We are fully dependent on the voluntary efforts of these experts to publish the journal for the academic community. Over the past year, *PLOS Genetics* relied on 654 associate and guest editors to manage the 2,474 articles submitted to the journal, which were assessed by nearly 3,000 reviewers. Their efforts enabled the publication of more than 680 rigorous, Open Access papers.

The names of our 2016 editors that handled submitted manuscripts appear in the Supporting Information as S1 Editor List and as S1 Guest Editor List. Our reviewers appear in the Supporting Information as S1 Reviewer List. We are grateful for their dedication, generosity, and support of Open Access science. Thank you all.

## Supporting information

S1 Editor List Alphabetical list of 2016 *PLOS Genetics* Editorial Board Members. (PDF)

S1 Guest Editor List Alphabetical list of 2016 *PLOS Genetics* Guest Editors. (PDF)

S1 Reviewer List Alphabetical list of 2016 *PLOS Genetics* peer reviewers. (PDF)
