# Supplementary material for: PLOS Genetics 2016 Reviewer and Editorial Board Thank You
Source: PLoS Genet. 2017 Mar 20;13(3):e1006671. doi: 10.1371/journal.pgen.1006671 (PMC5358728; doi:10.1371/journal.pgen.1006671)
Supplement: S1 Editor List — (PDF) [file pgen.1006671.s001.pdf]

PLOS | GENETICS

PLOS Genetics would like to thank all those who served on the Editorial Board in 2016:

Gonçalo Abecasis
A. Aziz Aboobaker
Mark Achtman
Julie Ahringer
Joshua Akey
David Allison
Kaveh Ashrafi
Utpal Banerjee
Gregory Barsh
Marisa Bartolomei
Adam Bass
John Bateman
David Begun
David Beier
Casey Bergman
Wendy Bickmore
Kirsten Bomblies
Giovanni Bosco
Jürgen Brosius
Christopher Brown
William Burkholder
Geraldine Butler
Josep Casadesús
Xuemei Chen
Vivian Cheung
Andrew Chisholm
Andrew Clark
Bruce Clurman
Gitta Coaker
Paula Cohen
Mónica Colaiácovo
Graham Coop
Gregory Cooper
Gregory Copenhaver
Chris Cotsapas
Gregory Cox
Claude Desplan
Anna Di Rienzo
Aimee Dudley
Susan Dutcher
Charis Eng
Michael Epstein
Justin Fay
Cédric Feschotte
Aleksandra Filipovska
Elizabeth Fisher
Jonathan Flint
Wayne Frankel
Matthew Freedman
Michael Freitag
Danielle Garsin
Ronald Gartenhaus
Greg Gibson
Takashi Gojobori
Dmitry Gordenin
John Greally
Henry Greely
Mathilde Grelon
H. Leighton Grimes
David Guttman
James Haber
Sarah Hake
Bruce Hamilton
Peter Hammerman
R. Scott Hawley
Lin He
Joseph Heitman
Hopi Hoekstra
Anita Hopper
Marshall Horwitz
Diarmaid Hughes
Kent Hunter
Sue Jinks-Robertson
Nicholas Katsanis
Daniel Kearns
Stuart Kim
Nicole King
Claudia Köhler
Artyom Kopp
Achim Kramer
Leonid Kruglyak
David Kwiatkowski
Tuuli Lappalainen
Nils-Göran Larsson
Suzanne Leal
Jeannie Lee
Tosso Leeb
Petra Levin
Michael Lichten
Ruth Loos

Bingwei Lu
Trudy Mackay
Hiten Madhani
Nancy Maizels
Harmit Malik
Susan Mango
Jonathan Marchini
Ivan Matic
Rodney Mauricio
John McDowell
Peter McKinnon
Michael McManus
Eric Miska
Ortrun Mittelsten Scheid
Cecilia Moens
Gloria Muday
Mary Mullins
Stefan Mundlos
Coleen Murphy
Amanda Myers
Michael Nachman
Joseph Opferman
Harry Orr
Bret Payseur
Christopher Pearson
Norbert Perrimon
Steven Petrou
Dmitri Petrov
Craig Pikaard
Vincent Plagnol
Sharon Plon
Jonathan Pritchard
Li-Jia Qu
Christine Queitsch
Wolf Reik
Paul Richardson
Samuli Ripatti
Derry Roopenian
Susan Rosenberg
Mikkel Schierup
Patrick Schnable
Robert Schneider
Liliane Schoofs
Nicholas Schork
Dirk Schübeler
Hamish Scott
Jeff Sekelsky
Licia Selleri
Tricia Serio
Nadia Singh
Giorgio Sirugo
Michael Snyder
Lotte Søgaard-Andersen
Nancy Spinner
Nathan Springer
Barbara Stranger
Lisa Stubbs
Eva Stukenbrock
Yousin Suh
Beth Sullivan
Lorraine Symington
Paul Taghert
Man-Wah Tan
Hua Tang
Sarah Tishkoff
David Toczyski
Bas van Steensel
Patrick Viollier
Peter Visscher
Hongyan Wang
Christine Wells
Emma Whitelaw
Andrew Wilkie
Scott Williams
Michael Worobey
Hao Yu
Mihaela Zavolan
Eleftheria Zeggini
Jianzhi Zhang
Jin-Qiu Zhou
