# Supplementary material for: PLOS Genetics 2016 Reviewer and Editorial Board Thank You
Source: PLoS Genet. 2017 Mar 20;13(3):e1006671. doi: 10.1371/journal.pgen.1006671 (PMC5358728; doi:10.1371/journal.pgen.1006671)
Supplement: S1 Guest Editor List — (PDF) [file pgen.1006671.s002.pdf]

PLOS Genetics would like to thank all those who served as a Guest Associate Editor in 2016:

Brett Abrahams
Devin Absher
Ian Adams
Andrés Aguilera
Juan Alfonzo
J. Andrew Alspaugh
Shoshy Altuvia
Leif Andersson
Alex Andrianopoulos
Mireille Ansaldi
Francisco Antequera
Jesus Aparicio
Heinz Arnheiter
Irina Artsimovitch
Ruth Ashery-Padan
Themistocles Assimes
Martin Augsten
Jeffrey Axelrod
Catherine Bachewich
Alexander Badyaev
Allan Balmain
Frederic Bantignies
Diana Baralle
Scott Barolo
Rodolphe Barrangou
Rowan Barrett
Antoni Barrientos
Nick Barton
Renata Basto
Cynthia Beall
Mark Beaumont
Hugo Bellen
Jennifer Benanti
Philip Benfey
Derk ten Berge
Tomas Bergström
Esther Betran
Marco Bianchi
Anja-Katrin Bielinsky
Mark Biggin
Brad Binder
Serge Birman
Douglas Bishop
Seth Blackshaw
Anne Blangy
Jesse Bloom
Kerry Bloom
Justin Blumenstiel
Rolf Bodmer
Vladimir Botchkarev
Adam Boyko
Axel Brakhage
Dana Branzei
Rachel Brem
Myles Brown
Sharon Browning
Anne Brunet
Maja Bucan
Sandeep Burma
William Bush
Ken Cadigan
Rafael Daniel Camerini-Otero
Judith Campbell
Xinwei Cao
Blanche Capel
Lucia Carbone
Miguel Carneiro
Agamemnon Carpousis
Antony Carr
Tamara Caspary
Amy Caudy
Eric Chang
Demian Chapman
Daniel Chasman
Ian Cheeseman
Jin Chen
Ping Chen
Wei Chen
Alice Cheung
Takahiro Chihara
Judy Cho
Surinder Chopra
Cheng-Ming Chuong
Mete Civelek
Leigh Clark
Sarah Cobey
Orna Cohen-Fix
Douglas Cook
Tiffany Cook
Tim Cooper

Vaughn Cooper
Justin Cotney
Justin Courcelle
Dana Crawford
Chad Creighton
Peter Crino
Damien D'Amours
Jennifer Darnell
Dean Dawson
Dennis Dean
Jurrien Dean
Eros Denchi
Wu-Min Deng
Megan Dennis
Sharon Dent
Xavier Didelot
José Dinneny
Victor DiRita
Christine Disteche
Ian Dodd
W. Ford Doolittle
Bruce Draper
Dennis Drayna
Tom Druet
Li-Lin Du
Andrea Duina
Sarah Dunstan
Scott Edwards
Todd Edwards
Sherif El-Khamisy
Sandra Encalada
Mark Estelle
Xavier Estivill
Eduardo Eyras
Adam Eyre-Walker
Daniel Falush
Gang Fang
Christian Fankhauser
Lindsay Farrer
Marcus Feldman
Xiaoqi Feng
Jonathan Filée
Brian Finck
Ruth Finkelstein
Judith Fischer
Jiri Forejt
Christine Foyer
Michael Freeling
Thomas Friedman
Rebecca Fry
Kimberly Gallagher
Nicolas Galtier
Fen-Biao Gao
Richard Gardner
David Gatfield
Brandon Gaut
Jennifer Gerton
Nick Gilbert
Thomas Giordano
Santhosh Girirajan
Anna Gloyn
Mary Goll
Joel Goodman
Miriam Goodman
Lisa Goodrich
Anne Goriely
Myriam Gorospe
Kathleen Gould
Michael Granato
Simon Gravel
Joost Gribnau
Jacques Grill
Martien Groenen
Rita Gross-Hardt
Ingrid Grummt
Frank Grützner
Jonathan Haines
Petra Hajkova
Robert Haltiwanger
Zhe Han
Mary Ann Handel
James Hane
Maureen Hanson
J. Marie Hardwick
Brian Harfe
Lea Harrington
Steven Harris
Nicholas Hastie
Christopher Hayes
Denis Headon
Jean Hébert
Erin Heinzen
Ian Henderson
Brian Hendrich
Clarissa Henry
Elise Héon
Ryan Hernandez
Michal Hershfinkel
Wolf-Dietrich Heyer
John Hickey
Alan Hinnebusch
Kendal Hirschi

Eva Hoffmann
Thomas Hoffmann
Robert Hofstra
Marina Holz
Kelly Hughes
Steve Humphries
Brian Huntly
Enamul Huq
Michael Ibba
Alexander Idnurm
Akihiro Ikeda
Kenneth Irvine
Sudha Iyengar
Vishwanath Iyer
Ian Jackson
Katherine Janeway
Heinrich Jasper
Sue Jaspersen
Kai Jiao
Francis Jiggins
Peng Jin
Alexander Johnson
Mark Johnson
Jonathan Jones
Joan Jorgensen
Eric Jorgenson
David Kadosh
Julie Kadrmas
Klaus Kaestner
Sundeep Kalantry
Gary Karpen
Manfred Kayser
Brendan Keating
William Kelly
Imtiaz Khan
Jaegil Kim
Seung Kim
Veronica Kinsler
Kristina Kirschner
Hannah Klein
Daniel Kliebenstein
R. Frank Kooy
Raphael Kopan
Rudolf Korinthenberg
Helmut Kramer
Christa Kuehn
Anshul Kundaje
Edmund Kunji
Martin Kupiec
Albert La Spada
Joseph Lachance
Eric Lambie
Robert Landick
Laura Landweber
Erica Larschan
Jan Larsson
Lionel Larue
Michael Laub
Jozef Lazar
Beth Lazazzera
Judith Leatherman
Ben Lehner
Bruno Lemaitre
Michael Lenhard
Alan Leonard
Thomas Leustek
Avraham Levy
Bingshan Li
Liming Li
Yi Li
Hong-Xuan Lin
Xihong Lin
Jeffrey Lipton
Michael Lisby
Ryan Lister
Barry London
Fanxin Long
Valter Longo
Bernard Lopez
Jennifer Loros
Christopher Lowe
Qing Lu
Tiegang Lu
Xiaowei Lu
Xin-Yun Lu
Erik Lundquist
James Lupski
Aldons Lusis
Henry Lynch
David Lyons
Hong Ma
Amy Maddox
Kateryna Makova
Carlo Maley
Francis Martin
Erika Matunis
Michael McMurray
Ruth McPherson
Barbara Mellone
Jane Mellor
Eric Mendenhall
Raphaël Mercier

Diane Merry
Philipp Messer
Mark Metzstein
Richard Michelmore
Jonathan Millar
Hyun Min Kang
Tom Misteli
Jennifer Mitchell
Kenneth Moberg
Kazufumi Mochizuki
Denise Montell
Stephen Montgomery
Bruce Morgan
Cynthia Morton
Ivan Moskowitz
Louis Muglia
Steven Munger
Sean Munro
Andrew Murray
Anna Murray
Jeremy Nance
Serge Nef
Aaron Neiman
Kim Nichols
Zachary Nimchuk
Michael Nitabach
Clarissa Nobile
Markus Noethen
Paul Northcott
Rebecca Oakey
Moira O'Bryan
David Oppenheimer
Terry Orr-Weaver
Henrik Oster
Sarah Otto
Karolina Pajerowska-Mukhtar
Leo Pallanck
Subba Palli
Martin Parniske
Tanya Paull
Aimee Payne
Zhen-Ming Pei
Sarah Pendergrass
Melissa Pepling
Matthias Peter
Antoine Peters
Joshua Plotkin
R. Scott Poethig
Barry Pogson
Katherine Pollard
Michael Polymenis
Daven Presgraves
Jeffrey Price
Molly Przeworski
Marcel Quint
David Raible
Kristijan Ramadan
John Rathjen
Amir Rattner
Timothy Reddy
Jason Reed
David Reich
Stephane Richard
JoAnne Richards
Lynn Riddiford
Karel Riha
Manuel Rivas
Steven Roberts
Antonis Rokas
David Ron
Vicki Rosen
Julian Sale
Ernesto Sánchez-Herrero
Michael Scanlon
Zach Schafer
Helgi Schioth
Markus Schmid
Jeremy Schmutz
Danny Schnell
Julian Schroeder
Nikolaus Schultz
Richard Schultz
Cordula Schulz
Yuri Schwartz
Ole Seehausen
Guy Sella
Vijay Setaluri
Konstantin Severinov
Anna Sheppard
Gavin Sherlock
Jianxin Shi
David Shore
Mark Shriver
Mark Siegal
Lyle Simmons
Robert Sladek
R. Keith Slotkin
Harold Smith
Marcus Smolka
Nahum Sonenberg
Thomas Spencer
Daniel St Johnston

Gary Stacey
Jason Stajich
Angelike Stathopoulos
Derek Stemple
Matthew Stephens
Susan Suarez
Venkatesan Sundaresan
Shamil Sunyaev
Peter Swoboda
Shahragim Tajbakhsh
Dan Tawfik
Martin Taylor
David Teis
Kathrin Thedieck
Pascal Therond
Timothy Thornton
Andreas Thum
Carl Thummel
Keiko Torii
Maria-Elena Torres-Padilla
Paul Trainor
Aleksandra Trifunovic
Heather True
Miltos Tsiantis
Renée Tsolis
Mick Tuite
Paul Turner
Jouni Uitto
Ioannis Vakonakis
Eliezer Van Allen
David Van Vactor
Patrick Varga-Weisz
Reiner Veitia
Marie-Hélène Verlhac
Pablo Visconti
Jörg Vogel
Tom Vogt
Benjamin Voight
Paul Wade
Claes Wahlestedt
Marian Walhout
Graham Walker
Douglas Wallace
John Wallingford
Jörn Walter
Guo-Liang Wang
Jue Wang
Shuang Wang
Yingxiang Wang
Xing Wang Deng
Matthew Warman
Masakatsu Watanabe
Matthew Webster
Robert Weinberg
Seth Weinberg
Daniel Weinberger
Ronit Weisman
James Wells
David Whitmore
Hans Widlund
Janey Wiggs
Tim Wiltshire
Christian Wolfrum
Francis-André Wollman
Richard Wood
Doris Wu
Ting Xie
Allison Xu
Wei Yan
Jing Yang
Yingzi Yang
Humphrey Yao
Noah Zaitlen
Kang Zhang
Pumin Zhang
Hongyu Zhao
Huilin Zhou
Xiaofeng Zhu
Manuel Zimmer
Jian Zuo
Laurence Zwiebel
