# Supplementary material for: PLOS Genetics 2016 Reviewer and Editorial Board Thank You
Source: PLoS Genet. 2017 Mar 20;13(3):e1006671. doi: 10.1371/journal.pgen.1006671 (PMC5358728; doi:10.1371/journal.pgen.1006671)
Supplement: S1 Reviewer List — (PDF) [file pgen.1006671.s003.pdf]

PLOS Genetics would like to thank all those who reviewed on behalf of the journal in 2016:

Alejandro Aballay
Ali Abbasi
Fatthy Abdel Tawab
Steffen Abel
Hermann Aberle
Mark Abney
Brett Abrahams
Devin Absher
Susan Ackerman
Brian Ackley
Viktor Adalsteinsson
Mark Adams
David Adams
Ian Adams
Reuven Agami
Aneil Agrawal
Pablo Aguilar
Andrés Aguilera
Nadav Ahituv
Bungo Akiyoshi
Eric Alani
Qais Al-Awqati
Frank Albert
David Albertini
Urs Albrecht
Juan Alfonso
Nicola Allen
Benjamin Allen
Laura Almasy
Scott Alper
Victor Ambros
Angelika Amon
Guy Amster
Anna Amtmann
Tim Anderson
Matthew Anderson
Carol Anderson
Göran Andersson
Kanae Ando
Noemi Andor
Ole Andreassen
Eran Andrechek
Montserrat Anguera
Anne Pfeiffer
Stylianos Antonarakis
Oscar Aparicio
Bruce H. Appel
Luís Aragón
Hiroyuki Arai
Eli Arama
Michelle Arbeitman
Jack Arbiser
Simon Archer
John Aris
Dan Arking
Robert Arkowitz
Virginia Armbrust
Nicolas Arnaud
Cheryl Arrowsmith
Valder Arruda
Vadim Arshavsky
Mario Arteaga-Vázquez
Helen Arthur
Irina Artsimovitch
Atsushi Asakura
Tadao Asami
Miho Asaoka
Hugues Aschard
Orr Ashenberg
Motoyuki Ashikari
Zoe June Assaf
Gil Ast
Kenneth Aston
Amr Aswad
Didier Auboeuf
Anjon Audhya
Paul Auer
Steven Austad
Johan Auwerx
Javier Avalos
Philip Avner
Michael Axtell
Nagi Ayad
Willy Baarends
Paul Babitzke
Erika Bach
Andreas Bachmair
Ruxandra Bachmann
Klaus Badenhoop
Paul Badenhorst

Richard Baer
Charles Baer
Anindya Bagchi
Yong-Sun Bahn
Xiaochun Bai
John Baillie
Richard Baines
Stuart Baird
Scott Baird
Nicholas Baker
Mark Baker
Christopher Bakkenist
Guus Bakkeren
Jeroen Bakkers
David Balding
Daniel Balick
Ravi Balijepalli
David Baltrus
Amitabha Bandyopadhyay
Jillian Banfield
Claudia Bank
Frederic Bantignies
Flora Banuett
Zhirong Bao
Fernando Baquero
Francisco Baralle
Daniel Barbash
Welsey Barber
Lisa Barcellos
Allison Bardin
Menashe Bar-Eli
Naama Barkai
Alice Barkan
Jacqueline Barlow
Scott Barolo
Maureen Barr
Frédéric Barras
François-Xavier Barre
Luis Barreiro
Antoni Barrientos
Gregory Barsh
Nick Barton
Susan Baserga
Konrad Basler
Munira Basrai
Brenda Bass
Anne Bassett
Alexander Bassuk
Deepak Bastia
Analabha Basu
Basudha Basu
Gillian Bates
David Bates
Philip Bates
Brendan Battersby
Alexis Battle
Tuncay Baubec
Peggy Baudouin-Cornu
Stephanie Baulac
Lars Baumbusch
Helmut Bäumlein
Isabel Bäurle
Jonathan Baxter
Vafa Bayat
Elizabeth Bayne
Dennis Bazylinski
Katarzyna Bebenek
Emily Beck
Anke Becker
Kate Beckingham
Philip Becraft
Bertrand Bed'Hom
Gary Beecham
Avraham Be'Er
Traude Beilharz
Greg Beitel
Gill Bejerano
Simon Bekker-Jensen
Faith Belanger
William Belden
Hugo Bellen
Mathias Beller
Xavier Belles
Deborah Bell-Pedersen
Jennifer Benanti
Shay Ben-Aroya
Yoselin Benitez-Alfonso
Matthias Benoit
Yehuda Ben-Shahar
Smadar Ben-Tabou De-Leon
Sigal Ben-Yehuda
Jonathan Berg
Jeremy Berg
Frederic Berger
Alan Bergland
Andreas Bergmann
Judith Berman
Daniel Bernard
Kara Bernstein
David Bertioli
Andrea Betancourt
Mireille Betermier

Esther Betran
Needhi Bhalla
Shoumo Bhattacharya
Sharon Bickel
Stephanie Bielas
Anja-Katrin Bielinsky
Mariann Bienz
Ethan Bier
Leslie Biesecker
Piter Bijma
David Bilder
Albrecht Bindereif
Emanuele Biondi
Kenneth Birnbaum
Douglas Bishop
Anthony Bishopp
Julie Biteen
Brian Black
Andrew Blackford
T. Keith Blackwell
Stacy Blain
Seth Blair
Alexandre Blais
Jill Blankenship
Susan Blanton
Todd Blevins
Kerry Bloom
Julian Blow
Elizabeth Blue
Michael Blum
Justin Blumenstiel
Tom Blumenthal
Michael Boddy
Rolf Bodmer
Jef Boeke
Arjen Boender
Derek Boerboom
Laszlo Bogre
Dirk Bohmann
Stefan Böhringer
Brigitte Boizet-Bonhoure
Michael Boland
Ewelina Bolcun-Filas
Monique Bolotin-Fukuhara
Hanno Bolz
Roberto Bonasio
Gareth Bond
Nina Bonekamp
Amelie Bonnefond
Carsten Bonneman
Amanda Bonner
Dorret Boomsma
Charles Boone
Frank Booth
Guntram Borck
Seth Bordenstein
Ingrid Borecki
Lorenzo Borghi
Katherine Borkovich
Joerg Bormann
Anthony Borneman
Alex Bortvin
Neelanjan Bose
Mirte Bosse
Lionello Bossi
Vladimir Botchkarev
Natalie Botchkareva
Laura Botigue
Cortney Bouldin
Rebecca Boumil
Deborah Bourc'his
Guillaume Bourque
Bastien Boussau
Paola Bovolenta
Bruce Bowerman
Josephine Bowles
Grant Bowman
Adam Boyko
Yuri Bozzi
Thomas Brabbs
Shelton Bradrick
Clive Bramham
Marc Bramkamp
Ulrich Brandt
Gloria Brar
Ana Bratic
Robert Braun
Alexander Brehm
Rachel Brem
Jason Brickner
Miguel Brieno-Enriquez
Anne Britt
Neil Brockdorff
Susan Brockerhoff
Gudrun Brockmann
Peter Brodersen
Heather Broihier
Basil Brooke
David Brow
Grant Brown
Christopher Brown
Carolyn Brown

Sharon Browning
Martina Brueckner
Anne Brunet
Clifford Brunk
Michael Brunner
Vincent Bruno
Kasia Bryc
John Buchan
David Buchner
C. Robin Buell
Elena Buena-Gonzales
Alex Buerkle
Mariano Buffone
Silvia Bulfone-Paus
Hannes Bülow
Stephen Buratowski
Emanuele Buratti
Sean Burgess
Robert Burgess
Margit Burmeister
Lori Burrows
Vincent Burrus
Briana Burton
Alessia Buscaino
Marcus Buschbeck
William Bush
Allen Buskirk
Harmen Bussemaker
Laura Buttitta
Mark Buttner
Bruno Cadot
Qiuyin Cai
Joseph Calabrese
Dinis Calado
Luz Calderon Villalobos
Minal Caliskan
Patrick Callaerts
Mario Calus
John Calvert
Cinzia Calvio
Rafael Daniel Camerini-Otero
Shelagh Campbell
Gerard Campbell
Catarina Campbell
Jose Cancelas
Julie Canman
Barbara Cannon
Rita Cantor
Sharon Cantor
Xu Cao
Blanche Capel
Terence Capellini
Valeria Capra
Valerio Carelli
Örjan Carlborg
Teresa Carlomagno
Ana Carmena
Piero Carninci
Agamemnon Carpousis
Catherine Carr
João Carriço
Jason Carroll
Jeffrey Carroll
Russ Carstens
Margret Casal
Paula Casati
Patrick Casey
Tamara Caspary
Clare Casteel
Diego Castrillon
Alberico Catapano
Elena Cattaneo
Dominique Caugant
Gianpiero Cavalleri
Marcello Ceci
Maria Fernanda Ceriani
Anthony Cesare
Andrei Chabes
Eunyoung Chae
Yang Chai
Sreekanth Chalasani
Danny Chan
Guillaume Chanfreau
Howard Chang
Qiang Chang
Chenbei Chang
Sung-Hee Chang
Moses Chao
Lin Chao
Michael Chao
Matthew Chapman
Marika Charalambous
Julia Charles
Deborah Charlesworth
Carole Charlier
Daniel Chasman
Nilanjan Chatterjee
Dhruba Chattoraj
Shawn Chavez
Ian Cheeseman
Z. Jeffrey Chen
Xuemei Chen

Rujin Chen
Meng Chen
Ju Chen
Bin Chen
Lin Chen
Di Chen
Chia-Yen Chen
Hao Chen
Quan Chen
Guo-Bo Chen
Zhukuan Cheng
Chonghui Cheng
William Cheng
Stephen Chenoweth
Andrew Chess
Alice Cheung
James Cheverud
Chin Chiang
Cheng-Ting Chien
Rayan Chikhi
Patrick Chinnery
Ian Chin-Sang
Jerry Chipuk
Dan Chitwood
QueeLim Ch'ng
Ken Cho
Hyung-Taeg Cho
Kang Chong
Yves Choquet
Seemay Chou
Zhaohui Chu
Diana Chu
George Chuck
Dongjun Chung
Cheng-Ming Chuong
Edward Chuong
Olivier Cinquin
Rafal Ciosk
Brian Ciruna
Mete Civelek
Peter Claes
Steven Clapcote
Susan Clark
Amander Clark
Scott Clark
Leigh Clark
David Clark
Nathan Clark
Hugh Clarke
John Cleary
Thomas Cline
David Clouthier
Aurélie Cobat
John Cobb
J. Mark Cock
Marco Coelho
Maureen Coetzee
Ehud Cohen
Paul Cohen
Christopher Colbert
James Collins
Eric Collisson
Lucia Colombo
Luca Comai
Iñaki Comas
Gavin Conant
Paul Conduit
Elizabeth Conibear
Steve Conlan
Miguel Constancia
Marco Conti
Silvestro Conticello
Edwin Cook
Mark Cookson
Joseph Coolon
Thomas Cooper
Vaughn Cooper
Tim Cooper
Sara Cooper
Shelley Copley
Anita Corbett
Mark Corbett
Victor Corces
Heather Cordell
Valerie Cormier-Daire
Robert Cornell
Francois Cornet
Jason Corwin
Frank Costantini
Carlo Costantini
James Cotterell
George Coupland
Shay Covo
Leah Cowen
Michael Cox
Timothy Cox
Erin Cram
Robert Cramer
Nigel Crawford
Dana Crawford
Alexander Crawford
Bryan Crawford

Nick Crawford
Bernard Crespi
Steve Crews
Richard Cripps
Fred Cross
Sean Crosson
Nicholas Croucher
Carlos Cruchaga
Gage Crump
Györgyi Csankovszki
Rutao Cui
Joaquim Culi
Lisa Cunningham
M. Joan Curcio
James Curran
Sean Curran
Jack da Silva
Alain Dabdoub
Tal Dagan
Anupama Dahanukar
Richard Dahl
Christian Dahmann
Love Dalén
Weiwei Dang
Fabrice Danjou
Janine Danks
Gabriella D'Arcangelo
Sébastien Darras
Sapna Das-Bradoo
Vincent Daubin
Della David
Alan Davidson
Lance Davidson
Irwin Davidson
Neil Davies
Philip Davy
Igor Dawid
Suzanne Dawid
Alessandra d'Azzo
Subhajyoti De
Paul de Bakker
Mario de Bono
Jan-Willem de Gier
Cristina de Guzman Strong
Jesus de la Cruz
Nicholas De Lay
Alexandre de Lencastre
Gustavo de los Campos
Ruud de Maagd
Nicola De Maio
Arnaud De Muyt
F.J. de Sauvage
Rohan de Silva
Elizabeth De Stasio
Lieven De Veylder
Harold de Vladar
Niels de Wind
Michael Dean
Wim Declercq
Veronique Decroocq
Christoph Dehio
Filippo Del Bene
Juan Carlos del Pozo
Jean-Marie Delalande
Carolin Delker
Lauren Dembeck
Marcel Den Hoed
Wu-Min Deng
Xiuxin Deng
Deepti Deobagkar
Ronald Depinho
Bart Deplancke
Jean-Marc Deragon
Michael Desai
Karl Desch
Thierry Desnos
Alison Devonshire
Olivier Devuyst
Walter Dewitte
Eleftherios Diamandis
Xianmin Diao
Francisco Diaz
Christian Dibble
Dion Kai Dickman
Giorgio Dieci
Andreas Diepold
Christoph Dieterich
Stephen Difazio
Marian Difiglia
Andrew Dillin
Marcus Dillon
Stefan Dimitrov
Mei Ding
Zhaojun Ding
José Dinneny
Christine Disteche
Jürgen Dittmer
Michael J. Dixon
Julianne Djordjevic
Ron Do
Bradley Doble
Adam Dobson

Chris Doe
Dan Doherty
Juergen Dohmen
Gregoriy Dokshin
Esteban Domingo
Benjamin Domingue
Julie Donaldson
Juan Dong
Aiwu Dong
Christopher Donnelly
John D'Orazio
Thilo Dörk
Patricia Dos Santos
Jessica Downs
Dennis Drayna
Thomas Dresselhaus
Robert Drewell
Konstantinos Drosatos
Dongsheng Duan
Dharani Dubey
Manu Dubin
Henry Duff
Siobain Duffy
Gaurav Dugar
Priya Duggal
Guillaume Dumenil
Jay Dunlap
Malcolm Dunlop
Gary Dunny
Sally Dunwoodie
Adam Dupuy
Jean-Maurice Dura
Bénédicte Durand
Jörg Durner
Daniel Durocher
Monica Dus
Troy Duster
Julien Dutheil
Alex Duval
Dan Dykhuizen
Ashlee Earl
Gregory Ebel
Johann Eberhart
Julia Edgar
Bruce Edgar
Dan Ehninger
Ian Ehrenreich
Patrick Eichenberger
Steve Eichten
Eli Eisenberg
Avigdor Eldar
Nels Elde
Sarah Elgin
Montserrat Elías-Arnanz
Hans Ellegren
Jan Ellenberg
Craig Ellermeier
Ronald Ellis
Jeff Ellis
Leigh Ellis
Nathan Ellis
Steven Ellis
Taline Elmayan
Ezzat El-Sherif
Patrick Emery
Kazuo Emoto
Joanne Engebrecht
Joanne Engel
Kurt Engeland
Barbara Engelhardt
Bevin Engelward
Mark Enright
Sevinc Ercan
Albert Erives
Cagla Eroglu
Nilufer Ertekin-Taner
Mafalda Escobar
Yuval Eshed
Jeffrey Esko
Olivier Espéli
Mark Estelle
Dafydd Evans
Matthew Evans
David Evans
Jonathan Ewbank
Elena Ezhkova
Marc Robert Fabian
James M. Fadool
Birthe Fahrenkrog
Ahmed Faik
Neil Fairweather
Daniel Falush
Shengyun Fang
Shenying Fang
Yanshan Fang
Yun Fang
Charles Farber
Margarida Fardilha
David Fardo
Mario Fares
David Fargo
Jason Farrar

Sébastien Faucher
David Fay
Martha Fedor
Michael Feldbrügge
Marie-Anne Félix
Jacques Fellay
Tim Fenton
Vilaiwan Fernandes
Oscar Fernandez-Capetillo
Miguel Ferreira
Martin Ferris
Andrzej Fertala
Mike Fessing
Philine Feulner
Richard ffrench-Constant
Aretha Fiebig
Marie-Dominique Filippi
Peter Fineran
Steven Finkel
Ruth Finkelstein
Carrie Finno
Krista Fischer
Susan Fisher
Amanda Fisher
Rafael Fissore
Greg Fitzharris
David Fitzpatrick
Klas Flardh
Michael Flister
Bruno Fonseca
James Ford
Tim Formosa
Naomi Forrester
Susan Forsburg
Ian Forster
Mary Fortune
Elpida Fragouli
Claire Francastel
Claudio Franceschi
Michael Francis
Alison Frand
F. Chris Franklin
Roberta Fraschini
Hunter Fraser
Timothy Frayling
Ian Frayling
Thomas Freeman
Andrew French
Nir Friedman
Thomas Friedman
Maxim Frolov
Marcus Fruttiger
James Fry
Xiang-Dong Fu
Isabelle Fudal
Tatsuo Fukagawa
Ryuya Fukunaga
Jennifer Fung
Barbara Funnell
Eileen Furlong
Eva Furrow
Ryo Futahashi
Toni Gabaldon
Christopher Gabel
Jacek Gaertig
Daniel Gaffney
Fred Gage
Julien Gagneur
Paul Galardy
Jonathan Galazka
Miguel Gallach
Peter Gallant
Irene Gallego Romero
Maria-Trinidad Gallegos
Romain Gallet
Imed Gallouzi
Carlos Galvan-Ampudia
Fei Gao
Abel Garcia-Pino
Thomas Gardella
David Garfinkel
Gian Garriga
Anton Gartner
Jacob Garza
Charles Gasser
Walter Gassmann
Eric Gaucher
Kyle Gaulton
Mackenzie Gavery
Patricia J. Gearhart
Brian Gebelein
Jonathan Gent
Rita Gerardy-Schahn
Holger Gerhardt
Jeffrey Gerst
Rene Geurts
Milan Geybels
Amin Ghabrial
Anatole Ghazalpour
Arjumand Ghazi
Michel Gho
Shobha Ghosh

Debashis Ghosh
Mauro Giacca
Claudia Giambartolomei
Bryan Gibbon
Matthew Gibson
Robert Gifford
Yoav Gilad
Clement Gilbert
Lilach Gilboa
Matthew Gilliham
Reid Gilmore
Alexander Gimelbrant
Edward Giniger
Mark Ginsberg
James Giovannoni
Tatiana Giraud
Aaron Gitler
David Glass
N. Louise Glass
Dominique Glauser
Michael Glotzer
Thomas Glover
Mark Glover
Matthew Goddard
Michael Goddard
Sarah Goetz
Alastair Goldman
Mark Gomelsky
Zachariah Gompert
Pierre Gönczy
Zhizhong Gong
Josefa González
Antonio González
Acaimo González-Reyes
Lisa Goodrich
Vera Gorbunova
Christopher Gordon
Philip Gordts
Harald Goring
Gregor Gorjanc
Myriam Gorospe
Jonatha Gott
Monica Gotta
Susan Gottesman
Mark Goulian
Geneviève Gourdon
Pravitt Gourh
Trevor Graham
Ian Grainge
David Grainger
Hugo Gramajo
Muriel Grammont
Barth Grant
Niels Grarup
Peter Graumann
Simon Gravel
Stephen Gray
Ian Greaves
Andy Greenfield
Bryan Greenhouse
David Greenstein
Eric Greer
Christopher Grefen
Peter Gregersen
David Gresham
Anastasia Gridasova
Thomas Gridley
Julian Griffin
Julia Grimwade
Paul Grini
Karen Gripp
Anthony Griswold
Kathryn Grive
Elisabeth Grohmann
Natalia Gromak
Jörg Gromoll
Ilan Gronau
Philippe Gros
Christina Gross
Andy Groves
Stephan Gruber
Peter Gruber
Wesley Grueber
Christoph Grunau
Xun Gu
Fangyi Gu
Zhenglong Gu
Vincent Guacci
Xin-Yuan Guan
Min-Xin Guan
Alba Guarné
Raul Narciso Guedes
Fang-Qing Guo
Yan Guo
Kyle Gustafson
Hubertus Haas
James Haber
Lilach Hadany
Alex Hajnal
Marc Halfon
Ruth Hall
Martin Hallberg

Mary Halloran
Eran Halperin
Hiroshi Hamada
Virginie Hamel
Marc Hammarlund
Molly Hammell
Thomas Hammond
Randy Hampton
Buhm Han
Guan-Zhu Han
Lynn Hancock
Mary Ann Handel
Alfred Handler
Courtney Hanna
Kasper Hansen
Anders Hansen
Maureen Hanson
Paul Hardin
Amnon Harel
Richard Harland
Frank Harmon
Steven Harris
Ann Harris
Kelley Harris
Charles Harris
Douglas A. Harrison
Christine Harrison
Rasika Harshey
Rune Hartmann
Rasmus Hartmann-Petersen
Natasha Harvey
Caroline Harwood
Gal Haspel
Terry Hassold
Nicholas Hastie
Paul Hasty
Ronald Hause
Alan Hauser
Susanne Häussler
Michael Hawrylycz
Christopher Hayes
Cole Haynes
Alex Hayward
David Hazlerigg
Chuan He
Ping He
Xin-Jian He
Chad He
Denis Headon
Rebecca Heald
Madhuri R. Hedge
Robert Hegele
Jayne Hehir-Kwa
Stefanie Heilmann
Max Heiman
Sven Heinz
Erin Heinzen
Carl-Philipp Heisenberg
James Hejtmancik
Yrjo Helariutta
Chris Helliwell
Gibran Hemani
Martin Hemberg
Ian Henderson
David Hendrixson
Corneliu Henegar
Regine Hengge
Sivan Henis-Korenblit
Brenna Henn
Clarissa Henry
Jamie Henzy
Nouria Hernandez
Elisabeth Herniou
Luis Herrera-Estrella
Hanspeter Herzel
Stephan Herzig
Erica Herzog
Rex Hess
Martin Hetzer
Patrick Heun
Wolf-Dietrich Heyer
Evelyne Heyer
Meleah Hickman
Ian Hickson
Robin Hiesinger
Philip Hieter
N. Patrick Higgins
Penelope Higgs
William Hill
Kent Hill
Robert Hill
Angie Hilliker
Axel Hillmer
Gary Hime
Hyouta Himeno
Justin Hines
Kevin Hiom
Takashi Hirayama
Yasushi Hiromi
Candice Hirsch
Heribert Hirt
Seiji Hitoshi

Christophe Hitte
Eileen Hoal
Asger Hobolth
Karin Hochrainer
Andreas Hochwagen
Andrea Hodgins-Davis
Jonathan Hodgkin
Alan Hodgkinson
Charles Hoffman
Eva Hoffmann
Kay Hofman
Michael Hofreiter
Deborah Hogan
Ben Hogan
Christer Hogstrand
Barbara Hohn
Matthew Holden
David Holding
Jay Hollick
Nancy Hollingsworth
William Holloman
Mark Holmes
Erik Holmqvist
Ben Holt
Saul Honigberg
Tomoaki Horie
Alexander Horswill
Steve Horvath
Arthur Horwich
Steven Hou
Xingliang Hou
Jianghui Hou
Douglas Houston
Alain Hovnanian
Martin Howard
Peter Howley
Ao-Lin Hsu
Hwei-Jan Hsu
Yen-Ping Hsueh
Cheng Hu
Yuxin Hu
Sui Huang
Shanjin Huang
Xun Huang
Rongfeng Huang
Linda Huang
Jirong Huang
Gang Huang
Hailiang Huang
Ru-Ting Huang
Aurélie Hua-Van
Jane Hubbard
Jim Hughes
Stacie Hughes
Kevin Hughes
John Huguenard
Andrew Huh
Jerome Ho Lam Hui
Peter Huijser
Timothy Humphrey
Steven Hunt
Patricia Hunt
Tony Hunter
John Huntriss
Enamul Huq
Daniel Hurley
Deborah Hursh
Greg Hurst
Anna Huttenlocher
Jean-René Huynh
Pirro Hysi
Alexander Idnurm
Masahito Ikawa
Hae Kyung Im
Yuzuru Imai
Takato Imaizumi
Jean-Luc Imler
Roger Innes
Ken Inoki
Michael Inouye
Dimitrios Ioannou
Iuliana Ionita-Laza
Ivan Iossifov
Florencia Irigoín
Vivian Irish
Alan Irvine
Meredith Irwin
Norio Ishida
Yuval Itan
Toshiro Ito
Koreaki Ito
Masaki Ito
Hiroshi Iwasaki
Ian Jackson
Eveliina Jakkula
Johan Jakobsson
Hsieh James
Guilhem Janbon
Gert Jansen
Lars Jansen
Verena Jantsch
Artur Jarmolowski

Dan Jarosz
Heinrich Jasper
Sue Jaspersen
Matti Jauhiainen
Patrick Jay
James B. Jaynes
Penny Jeggo
Albert Jeltsch
Dagan Jenkins
Paul Jenkins
Jeffrey Jensen
Kirk Jensen
Joe Jerry
Andrzej Jerzmanowski
Jenna Jewell
Songtao Jia
Jin Jiang
Huaqi Jiang
Rulang Jiang
Duo Jiang
Yongqin Jiao
Francis Jiggins
Peng Jin
Suk-Won Jin
Marek Jindra
Josef Jiricny
Mark Jobling
F. Bradley Johnson
Erik Johnson
Philip Johnson
Patricia Johnson
Jukka Jokela
Louise Jones
Chris Jones
Peter Jones
Felicity Jones
Thomas Jongens
Marcel Jonkman
Philip Jordan
Daniel Jordan
Eric Jorgenson
Emily Josephs
Gabor Juhasz
Han-Sung Jung
Ivan Juric
Andreas Kaczmarczyk
Sebastian Kadener
James Kadonaga
David Kadosh
Farid Kadyrov
Henrik Kaessmann
Daniel Kaganovich
Angela Kaindl
Tetsuji Kakutani
Krisztina Kaldi
Abhijit Kale
Alla Kalmykova
Auinash Kalsotra
Maria Kalyna
Sophien Kamoun
Malek Kamoun
Jörg Kämper
Harm Kampinga
Hunseung Kang
Aimee Kao
Craig Kaplan
Philipp Kapranov
Rachid Karam
Mariusz Karbowski
François Karch
Ted Karginov
Elinor Karlsson
Robert Karn
Yona Kassir
Judith Kassis
Riku Katainen
Tsutomu Katayama
Assaf Katz
Aris Katzourakis
Jay Kaufman
Karla Kaun
Liisa Kauppi
Timo Kauppila
John Kauwe
Kiyoshi Kawakami
Yasuhiko Kawakami
Tsutomu Kawasaki
Manfred Kayser
Jennifer Kearney
Jacob Keaton
Electron Kebebew
Jack Keene
Alex Keene
Scott Keeney
Kenneth Keiler
Erin Kelleher
Jonathan Keller
Ten-Hagen Kelly
Gavin Kelsey
Janet Kelso
Tony Kenna
Scott Kennedy

Linda Kenney
Ruth Keri
Oliver Kerscher
Rachel Kerwin
Javed Khan
Abderrahman Khila
Saadi Khochbin
Amy Kiger
James Kijas
Helena Kilpinen
Dennis Kim
Joomyeong Kim
John Kim
Seung Kim
Nayun Kim
Tae-Hee Kim
Tae-Min Kim
Mary-Claire King
Chris Kingswood
Tetsu Kinoshita
John Kirby
Viktor Kirik
Mark Kirkpatrick
Daiju Kitagawa
Toomas Kivisild
Lena Kjellen
Christian Klambt
Lisa Klasson
Alexander Kleger
Robert Klein
Daniel Kliebenstein
Gabriele Klug
Marie Kmita
Christopher Knight
Daren Knoell
David Knowles
Joshua Knowles
Karen Knudsen
Junya Kobayashi
Tatsuya Kobayashi
Daniel Koboldt
Thomas Kocher
Daniel Koenig
Kyunghee Koh
Annegret Kohler
Sepp Kohlwein
Terumi Kohwi-Shigematsu
Hisashi Koiwa
Anna Koltunow
Kiran Kondabagil
Nikolaos Konstantinides
Eugene Koonin
Charles Kooperberg
Günther Koraimann
Bruce Korf
Uwe Kornak
Daniel Kornitzer
Abraham Korol
Martin Korte
Tomokazu Koshiba
Douglas Koshland
Sanna Koskiniemi
Noora Kotaja
Ales Kovarik
Eiki Koyama
Julia Kozlitina
Lukasz Kozubowski
Peter Kraft
Helmut Kramer
David Krantz
Ksenia Krasileva
Michael Krause
Jordan Kreidberg
Martin Kreitman
Lumir Krejci
Wilhelm Krek
Hannie Kremer
Nevan Krogan
Lee Kroos
Joachim Krug
Sarah Kucenas
Ulrich Kück
Grzegorz Kudla
Ursel Kues
Kristina Kühn
Carol Kumamoto
Justin Kumar
Vivek Kumar
Anuj Kumar
Karl Kunert
Krushnamegh Kunte
Martin Kupiec
Hiroki Kurihara
Hidehito Kuroyanagi
Zoltán Kutalik
Andrei Kuzminov
Young Kwon
Michael Kyba
Charalambos Kyriacou
Albert La Spada
Soni Lacefield
Joseph Lachance

Raj Ladher
Michael Ladomery
Roosa Laitinen
Sarah Lambert
Dudley Lamming
Simon Lane
Gregory Lang
Chiara Lanzuolo
Louis Lapierre
Paul Lapointe
Oleg Laptenko
Rob Larkin
Germán Larriba
Erica Larschan
Lionel Larue
Janine Lasalle
Paul Lasko
Andrew Lassar
Brittany Lasseigne
Nelson Lau
On Sun Lau
Vincent Laudet
Patrizia Lavia
Martin Lavin
Matthew Law
Michael Lawrence
Brian Lazzaro
Tung Le
David Leach
Kyu-Sun Lee
Sang Eun Lee
Su-In Lee
Tzumin Lee
Michael Lee
Youngsook Lee
Grace Lee
Min Gyu Lee
Siu Lee
Seunggeun Lee
Seung-Jae Lee
Hsiu-Hsiang Lee
Seongsoo Lee
James Leebens-Mack
Véronique Lefebvre
Paul Lefebvre
Christopher Lefèvre
Jean-Luc Legras
Gaelle Legube
Michael Lehmann
Ulrich Lehmann
Ben Lehner
Elissa Lei
Lei Lei
Sebastian Leidel
Sergey Leikin
Jeff Leips
Bruno Lemaitre
Bernardo Lemos
Christopher J. Lengner
Ernst Lengyel
Thomas Lenormand
Alan Leonard
Thierry Lepage
Emmanuelle Lerat
Holger Lerche
Christina Leslie
Elizabeth Leslie
Laura Lettice
Guillaume Lettre
François Leulier
Richard Leventer
Mitchell Paul Levesque
Bruce Levin
Tera Levin
Mia Levine
Jean-Pierre Levraud
Avraham Levy
Kim Lewis
Peter Lewis
Ottoline Leyser
Tiansen Li
Liming Li
Yi Li
Chuanyou Li
Yunhai Li
Qi-Jing Li
Mingfa Li
Ling Li
Xin Li
Yun Li
Hongju Li
Ning Li
Sheena Li
Melody Li
Yanjing Li
Christine Li
Han Liang
Romain Libbrecht
Giordano Liberi
Andrew Lidral
Toby Lieber
Tami Lieberman

Susan Liebman
Robert Lightowlers
Christopher D. Lima
Haifan Lin
Danyu Lin
Huawen Lin
Yao-Cheng Lin
Charles Lin
Cecilia Lindgren
Ariel Lindner
Sara Lindström
Brian Link
Damon Lisch
Gianni Liti
Amy Litt
Tom Little
Yi Liu
Yaoguang Liu
Haoping Liu
Nianjun Liu
Karen Liu
Dajiang Liu
Yusen Liu
Yongzhong Liu
Hongtao Liu
Yilun Liu
Liang Liu
Jiang Liu
Jianmin Liu
Andy Liwang
Ana Llopart
Xavier Llor
Bertrand Llorente
Matxalen Llosa
Robert Lloyd
Alison Lloyd
Anders Løbner-Olesen
Lawrence Loeb
Robbie Loewith
Malcolm Logan
John Logsdon
Jan Lohmann
Kirk Lohmueller
Fanxin Long
Anthony Long
Henry Long
Javier Lopez
Miguel López
Luis Lopez-Molina
Jose Lopez-Ribot
Javier Lopez-Rios
Esben Lorentzen
Alexander Lorenz
Oscar Lorenzo
Zdravko Lorkovic
Christian Lorson
Susan Lott
Stephen Loughran
Edward Louis
Susan Lovett
Rui Lu
Qing Lu
Hua Lu
Xiaowei Lu
Qiongshi Lu
Bingwei Lu
Francesca Luca
Reini Luco
Neal Lue
Karolin Luger
Scott Lujan
Ed Luk
Brian Luke
Anders Lund
Erik Lundquist
Shishi Luo
Wenqin Luo
James Lupski
Arthur Lustig
Donald Lyall
David Lydall
David Lynch
Vincent Lynch
Gholson Lyon
Karen Lyons
Liang Ma
Hong Ma
Yong-Chao Ma
Stefan Maas
Fabio Macciardi
Marcy MacDonald
Lesley MacNeil
Frank Madeo
Andreas Madlung
Morris Maduro
Keith Maggert
Paul Magwene
Ram Maharjan
Christopher Maher
Ari Pekka Mahonen
Eleanor Maine
William Mair

Pascal Maire
Amit Majithia
Ho Yi Mak
Christopher Makaroff
Carl Malchoff
Fransiska Malfait
Anna Malkova
Frédérick A. Mallette
Moises Mallo
Jacob Malone
Julin Maloof
J. Robert Manak
Pablo Manavella
Eugenio Mancera
Nicholas Mancuso
Giovanni Manfredi
Arya Mani
Jaan Männik
John Mansfield
Roberto Mantovani
M. Chiara Manzini
Junhao Mao
Elisabeth Marchal
Alan Marchant
Adrian Marchetti
Douglas Marchuk
Stevan Marcus
Gregory Marczynski
Andrei Mardaryev
Daniel Marenda
Francesca Mariani
Stephen Maricich
Mohan Marimuthu
Frederic Marion-Poll
David Mark Welch
Katharina Markmann
Luciano Marraffini
Alexander Marson
Adele Marston
Eliane Marti
Kelsey Martin
Jack Martin
James Martin
Joanna Martin
Simone Martinelli
German Martinez Arias
Antonio Martínez-Laborda
Enrique Martinez-Perez
Keri Martinowich
Cesar Martins
Rasmus Marvig
Christopher Marx
Hisao Masai
Thorsten Mascher
Ulrike Mathesius
Iain Mathieson
Michael Matise
Kunihiro Matsumoto
Midori Matsumoto
Ichiro Matsumura
Makoto Matsuoka
Aras Mattis
Erika Matunis
David Matus
Martin Matzuk
Cedric Maurange
Matthew Maurano
Helen May-Simera
Mona Mazaheri
Gérard Mazon
Rachid Mazroui
Carolyn McBride
David McCandlish
John McCarrey
Brooke McCartney
David McCauley
David McClay
Sarah McClelland
Rachel McCord
Joe McCormick
Kathleen McCoy
Pierre McCrea
Christopher McDevitt
David McFadden
James McGhee
Kelly McGowan
Lauren McIntyre
Kim McKim
Francis McNally
Elizabeth McPherson
Gavin McStay
Mitch McVey
Graham McVicker
Jennifer Meadows
Paul Medvedev
Richard Meehan
Heather Mefford
Joana Meier
Colin Meiklejohn
David Meinke
Joshua Mell
Victoria Meller

Cathryn Mellersh
Stephen Meltzer
Eric Mendenhall
Juan Mendez
Cristina Menni
Juanita Merchant
Karen Merchante
Raphaël Mercier
Houra Merrikh
Alexey Merz
Jessi Mester
Ravikanth Metlapally
Brian Metzger
Wouter Meuleman
Mark Meuth
Joel Meyer
Andreas Meyerhans
Benedicte Michel
Tam Mignot
Irene Miguel-Aliaga
Marco Milan
Pleasantine Mill
Jonathan Mill
Sarah Millar
Andrew Millar
Sean Millard
Samuel Miller
Kenneth Miller
Dana Miller
Webb Miller
Kyle Miller
Craig Miller
Michal Minczuk
Alex Minella
Olivo Miotto
Mario Mirisola
Sergei Mirkin
Dan Mishmar
Tom Misteli
Aaron Mitchell
David Mitchell
Brian Mitchell
Phil Mitchell
Tanja Mittag
Masayuki Miura
Shuhei Miyashita
Kota Mizumoto
Danesh Moazed
Kazufumi Mochizuki
Mauro Modesti
Calvin Mok
Darren Monckton
M. Monje
David Monk
Kelly Monk
Magnus Monné
Dimitri Monos
Anne-Helene Monsoro-Burq
Jacques Montagne
Martin Montecino
Mervyn Monteiro
Denise Montell
Craig Montell
Stephen Montgomery
Kristi Montooth
Sally Moody
Lieve Moons
Sean Moore
Aixa Morales
Nancy Moran
Charles Moran
Kevin Morano
Alexandra Moreira
Roy Morello
Eduardo Moreno
Adrian Moreno
Celine Morey
Philip Morgan
Peter Morrell
Scott Morrical
Andrew Morris
Joachim Morschhäuser
Cynthia Morton
James Moseley
Richard Mott
Joseph Mougous
Joanna Mountain
Zissimos Mourelatos
Bernard Moussian
Loukas Moutsianas
Wellington Muchero
Gary Muehlbauer
Christoph Mueller
Saikat Mukhopadhyay
Carol Munro
Anne Murphy
Johanne Murray
John Murray
James Murray
Heath Murray
Jeremy Murray
Ville Mustonen

Günther Muth
Allen Myers
Kirk Mykytyn
Peter Myler
Angus Nairn
Toru Nakamura
Satoshi Namekawa
Jeremy Nance
Shima Naoko
Vivek Naranbhai
Maithreyi Narasimha
Arshan Nasir
Amy Navratil
Daniel Neafsey
Andreas Nebenführ
Richard Neher
Christoffer Nellaker
Christian Neri
Rob Ness
Ralph Neumüller
Jason Newbern
Carol Newlon
William Newman
Kim Newton
Joanne Ngeow
Ylan Nguyen
Ying Ni
Hannah Nicholas
Bryce Nickels
Alain Nicolas
Andreas Niebel
Conrad Nieduszynski
Jens Nielsen
Olaf Nielsen
Knud Nierhaus
Dietrich Nies
Timothy Niewold
Teruyuki Niimi
Hironori Niki
Yuri E. Nikiforov
Riko Nishimura
Isao Nishimura
Corey Nislow
Ryusuke Niwa
Dean Nizetic
Sumihare Noji
Ken-Ichi Nonomura
Mohamed Noor
Chris Norbury
Jared Nordman
Mariusz Nowacki
Romana Nowak
Vardis Ntoukakis
Dmitri Nusinow
Andre Nussenzweig
Sergey Nuzhdin
Todd Nystul
Jason O'Connor
Berl Oakley
Ma'en Obeidat
Moira O'Bryan
Donal O'Carroll
Michael O'Connor
Devin O'Connor
Marlene Oeffinger
Donata Oertel
Matthew Oetjens
Seishi Ogawa
Stacey Ogden
Tatsuya Ohhata
Uwe Ohler
Kinji Ohno
Raquel Oliveira
Brian Oliver
Sebastian Oltean
Masayuki Onishi
Itay Onn
Christiane Opitz
Michael O'Rand
László Orbán
Richard Ordway
Teresa Orenic
Eyleen O'Rourke
Guillaume Orsi
Kyle Orwig
Heinz Osiewacz
Mary Osley
Elaine Ostrander
Samir Ounzain
Tom Owen-Hughes
Edward Owusu-Ansah
Annalise Paaby
Maurizio Pacifici
Robert Page
Andrea Page-Mccaw
Kenneth Paigen
Eric Pailhoux
Kevin Painter
Coro Paisan-Ruiz
Javier Palatnik
Francesc Palau
Francesca Palladino

Leo Pallanck
Wei Pan
Udai Pandey
John Panepinto
Michael Pankratz
Sophie Pantalacci
Kai Papenfort
Francois Parcy
Peter Pare
Christopher Park
Roy Parker
Heidi Parker
Joel Parker
Miles Parkes
John Parkinson
Stephen Parnell
Matthew Parsek
Will Parsons
Mark Parthun
Terence Partridge
Leopold Parts
Bogdan Pasaniuc
Manijeh Pasdar
Philippe Pasero
Amy Pasquinelli
Maria Rita Passos-Bueno
Tomi Pastinen
Nipam Patel
Maulik Patel
Steve Paterson
Andrew Paterson
Sandeep Paul
Ralf Paus
Martin Pavelka
Graham Pavitt
Pavlos Pavlidis
Youri Pavlov
Wojciech Pawlowski
Gary Payne
Gregory Pazour
Cameron Peace
Jim Peacock
Ullas Pedmale
John Pehrson
York Pei
Mark Peifer
Stephan Peischl
Vladimir Pelicic
Gina Peloso
Jose Penades
Luiz Penalva
Alberto Pendas
Sarah Pendergrass
Jiajie Peng
Melissa Pepling
Caitlin Pepperell
Gislene Pereira
Christian Perez
Steven Perlman
Frédérique Peronnet
Laurent Perrin
John Perry
George Perry
Michael Perry
Benjamin Peter
Eva Petermann
Jason Peters
Jan-Michael Peters
Hilde Peters
Christian Petersen
Thomas Peterson
Thomas Petes
Ezequiel Petrillo
John Petrini
Dmitri Petrov
Slavé Petrovski
Vladislav Petyuk
Roberto Pezza
Boris Pfander
Alexander Pfeifer
Cathie Pfleger
Hemali Phatnani
Adam M. Phillippy
Michael Philpott
Tyler Picariello
Franck Pichaud
Curtis Pickering
Jonathan Pierce-Shimomura
Stuart Piertney
Vilja Pietiäinen
Francesca Pignoni
Craig Pikaard
Martin Pilhofer
Manoj Pillai
Zachary Pincus
Manuel Piñeiro
Lionel Pintard
Jeffrey Piotrowski
Roger Pique-Regi
Matti Pirinen
Jon Pittman
Antonius Plagge

Antonio Planchart
Christoph Plass
Serge Plaza
Jeffrey Pleiss
Anne Plessis
Jonathan Plett
Igor Pogribny
Christian Pohl
Paz Polak
Ozren Polasek
Richard Pomerantz
Kyle Pomraning
Frédéric Pontvianne
Anthony Poole
Aleksandar Popadic
Douglas Portman
Sean Post
Alex Postma
Wayne Potts
Francis Poulat
Joseph Powell
Veena Prahlad
Jai Prakash
Supriya Prasanth
Jill Preston
Mary Preuss
Jeffrey Price
Alkes Price
Clive Price
Nicholas Priest
Michael Prigge
David Prober
Daniel Promislow
Nick Proudfoot
Peter Pryciak
Zachary Pursell
Michael Purugganan
Manojkumar Puthenveedu
George Pyrowolakis
Wenfeng Qian
Hong Qiao
Feng Qiao
Udi Qimron
Genji Qin
Feng Qu
Peter Quail
David Quigley
Lluis Quintana-Murci
Melanie Quiver
Irfan Qureshi
Kimberly Raab-Graham
Fernando Rabanal
Leonard Rabinow
Catherine Rabouille
Sunish Radhakrishnan
Sabarinathan Radhakrishnan
David Raible
Kumaran Ramamurthi
Jorge Ramirez-Prado
Christiane Rammelt
Dale Ramsden
David Rand
Thomas Rando
Oliver Rando
Prashanth Rangan
Matthew Rasband
Mladen-Roko Rasin
David Rasko
Morten Rasmussen
Rajiv R. Ratan
William Ratcliff
Soumya Raychaudhuri
Cécile Raynaud
Donald Ready
Mark Rebeiz
Randy Rector
A. Hari Reddi
Peter Reddien
James Reecy
Randall Reed
Roger Reeves
Birgitte Regenberg
Heidi Rehm
Atteeq Rehman
Andreas Reichert
Chris Reid
Josephine Reinhardt
Valerie Reinke
Tânia Reis
Jeremy Reiter
Rainer Renkawitz
Natalia Requena
Kyle Retterer
Gunter Reuter
Rodrigo Reyes Lamothe
Mikael Rhen
Christa Rhiner
Lluís Ribas De Pouplana
William Rice
Daniel Rice
Peter Richard
Eric Richards

Helen Richardson
Stephen Richmond
Ellizabeth Rideout
Arne Rietsch
Jason Rihel
Filippo Rijli
Bruce Riley
Paul Riley
Vera Rinaldi
Niels Ringstad
Victoria Risbrough
Makarand Risbud
Manuel Rivas
Benjamin Roa
Silke Robatzek
Mark Roberson
Thomas Robert
Charles Robin
David Robinson
Gene Robinson
Rebecca Robker
Sonia Rocha
Christian Rocheleau
Matthew Rockman
Enrique Rodriguez-Boulan
Henry Roehl
Gregory Rogers
Jung Roh
Jean-Yves Roignant
Antonis Rokas
Filip Rolland
Casey Romanoski
Pascale Romby
Tony Romeo
Ze'ev Ronai
Lars Ronnegard
Jeanne Ropars
Joaquim Ros
Alan Rose
Mark Rose
Adam Rosebrock
Gil Rosenthal
James Rosinski
Eric Ross
Jeffrey Ross-Ibarra
Laura Rossini
Monica Roth
Siegfried Roth
Scott Rothbart
Lawrence Rothblum
Rodney Rothstein
Agnès Rotig
Philippe Roux
François Rouyer
Benjamin Rowland
Jesse Rowley
Sudipto Roy
Scott Roy
Peter Roy
Nikolay Rozhkov
David Rudner
Elena Rugarli
Davide Ruggero
José Ruiz-Herrera
Andres Ruiz-Linares
Steven Russell
Rick Russell
Jared Rutter
Guy Rutter
Ilya Ruvinsky
Joske Ruytinx
Robert Ryan
Hyung Don Ryoo
Paolo Sabelli
Giuseppe Saccone
Kirsten Sadler Edepli
Takashi Sado
Julio Saez-Vasquez
Alvaro Sagasti
Jørn Sagen
Supriya Saha
Jason Sahl
Shigeaki Saitoh
Daisuke Sakai
Yoichi Sakata
Julian Sale
Iris Salecker
Emili Saló
Nathan Salomonis
Joshua Sampson
Leona Samson
David Samuelson
Linda Sandell
Suzanne Sandmeyer
Tzu-Kang Sang
Rafael Sanjuán
Sriram Sankararaman
Vittorio Sartorelli
Masamitsu Sato
Yoko Satta
Glen Satten
Karin Sauer

Isabel Saur
Cathy Savage-Dunn
Pierre Savagner
Pierre Savatier
Ahilya Sawh
Peter Scacheri
Aylwyn Scally
Enrico Scarpella
Roel Schaaper
Stephen Schaffner
G. Eric Schaller
Christopher Schardl
Tim Schedl
Henrik Scheller
John Schiefelbein
John Schimenti
Karen Schindler
Ernestina Schipani
Jan Schirawski
Jennifer Schisa
Christine Schlacher
Bettina Schmid
Kristina Schmidt
Robert Schmitz
James Schnable
David Schneider
Kay Schneitz
Frank Schnorrer
Thorsten Schnurbusch
Sarah Schott
Julian Schroeder
Anthony Schryvers
Oren Schuldiner
Hinrich Schulenburg
Alan Schulman
Richard Schultz
Molly Schumer
Erwin Schurr
Thomas Schwartz
Robert Schwartz
François Schweisguth
Bianca Sclavi
Ralph Scully
Charles Scutt
Patrick Seale
Ole Seehausen
Pavel Seeman
David Segal
Nava Segev
Laure Segurel
Stephan Seiler
Rajandeep Sekhon
Michael Seldin
Ekaterina Semenova
Hiroshi Seno
Hak Soo Seo
Cathal Seoighe
Chetan Seshadri
Ane Sesma
A. Mark Settles
Konstantin Severinov
Thomas Sexton
Agnel Sfeir
Orie Shafer
Jagesh Shah
Shai Shaham
Michael Shapira
Igor Sharakhov
Shyam Sharan
Michael Sheehan
Val Sheffield
Samuel Shelburne III
Roger Sher
Michael Sherman
David Sherwood
Sanjay Shete
Qinghua Shi
Xiaobing Shi
Joseph Shieh
Sagiv Shifman
Ben-Zion Shilo
Sebastian Shimeld
Lawrence Shimkets
Kazuhiro Shiozaki
Dorothy Shippen
Assia Shisheva
David Shore
Mark Shriver
Jayendra Shukla
Michael Shy
Lyudmila Sidorenko
Derek Sieburth
Kellee Siegfried
Stephan Sigrist
Olin Silander
Alcino Silva
Neal Silverman
Lyle Simmons
Noah Simon
Kai Simons
Gordon Simpson
Don Sin

Andrew Sinclair
Ajeet Singh
Larry Singh
Sonal Singhal
Andrew Singleton
Neelima Sinha
Pradip Sinha
Rita Sinka
Haruhiko Siomi
Linda Siracusa
Kanishka Sircar
Per Sjödin
James Skeath
Daniel Skelly
Ahna Skop
Jan Skotheim
Efthimios Skoulakis
Jon Slate
Montgomery Slatkin
Matthew Slattery
Meghan Slean
R. Keith Slotkin
Gerald Smith
Chris Smith
Lisa Smith
Darren Smith
Rachel Smith-Bolton
Evan Snitkin
Elizabeth Snyder
Nara Sobreira
Kee Hoon Sohn
Nick Sokol
Evgeni Sokurenko
Eric Soler
Matthias Soller
Irina Solovei
David Somers
Yun Song
Wayne Sossin
Abdenour Soufi
Alexandra Soukup
Rita Sousa-Nunes
Julie Soutourina
Adam Sowalsky
Edgar Spalding
David Spector
Terence Speed
Doug Speed
Sabrina Spencer
Pietro Spitali
Steven Spoel
Henriet Springelkamp
Michael Springer
Richard Spritz
Jagan Srinivasan
Gary Stacey
Franklin Stahl
Maike Stam
Pamela Stanley
Jeremy Stark
Michelle Starz-Gaiano
Brian Staskawicz
Leonidas Stefanis
Catherine Stein
Eirikur Steingrimsson
Lars Steinmetz
Jorg Stelling
Deborah Stenkamp
Paul Sternberg
Oswald Steward
Ruth Steward
Alexandre Stewart
James Stewart
Rodney Stewart
Adrie Steyn
Bangyan Stiles
David Stillman
Hugo Stocker
Sophia Stone
Mark Stoneking
Jay Storz
Rolf Stottmann
Lucia Strader
Jeffrey Streelman
Andrea Streit
Lena Ström
Jörg Stülke
Jason Stumpff
Richard Sturm
Tin Tin Su
Le Su
Vijayalakshmi Subramanian
Henry Sucov
Peter Sudmant
Katsunori Sugimoto
Jose Suja
Jae Hoon Sul
Charlotte Sumner
Yan Sun
Wei Sun
Lei Sun
Jianjun Sun

Sibum Sung
Per Sunnerhagen
Hanna Susi
Keiichiro Susuki
Ann Sutherland
Mark Sutton
Hitomi Suzuki
John Svaren
Clive Svendsen
Petr Svoboda
Maurice Swanson
Dylan Sweetman
Andrea Sweigart
Heven Sze
Lóránt Székvölgyi
Moriah Szpara
Kikue Tachibana-Konwalski
Ioanna Tachmazidou
Yasuomi Tada
Hideki Takahashi
Kei-ichi Takata
Shunichi Takeda
Shohei Takuno
William Talbot
Michael Talkowski
Manuel Talón
Keiji Tanaka
Kun Tang
Dingzhong Tang
Wei-Hua Tang
Yuije Tang
Toshiyasu Taniguchi
Hiromu Tanimoto
Milos Tanurdzic
Ran Tao
Marco Tartaglia
Kei Tashiro
Kelly Tatchell
Diethard Tautz
Dan Tawfik
Deanne Taylor
Louis Taylor
Thomas Tedder
Aurelio Teleman
Marina Telonis-Scott
Daniel Tenen
Ron Tepper
Pilar Testillano
Cory Teuscher
Martin Thanbichler
Johan Thevelein
David Thomas
Leslie Thompson
Barry Thompson
Stefan Thor
Kai Thormann
Joseph Thornton
Timothy Thornton
Timothy Thurman
Thorsten Thye
Bin Tian
Irene Tiemann-Boege
Peter Tiffin
Marcel Tijsterman
Lubov Timchenko
Joanna Timmins
David Ting
Laurent Tiret
Vladimir Titorenko
Vijay Tiwari
David Tobin
John Todd
Seiichi Toki
Shubha Tole
Seth Tomchik
Kazuhito Tomizawa
Ian Tomlinson
Ivan Topisirovic
Miguel Torres
Victor Torres
Attila Toth
Aminata Toure
John Tower
Paul Trainor
Walther Traut
Ana Traven
Jessica Treisman
David Tremethick
Aleksandra Trifunovic
Felix Tropf
Ala Trusina
Nien-Pei Tsai
Betty Tsao
Hirokazu Tsukaya
Toshio Tsukiyama
Simon Tuck
Taru Tukiainen
Elizabeth Tunbridge
Anders Tunlid
Franziska Turck
Leslie Turner
Brett Tyler

Hiroki Ueda
Masaru Ueno
Mirka Uhlirova
Igor Ulitsky
Elçin Ünal
Nobuyuki Uozumi
Lawrence Uricchio
Thomas Vaccari
Hassan Vahidnezhad
Vito Valiante
Martijn van de Bunt
Yves van de Peer
Pim van der Harst
Esther van der Knaap
Louise van der Weyden
Marjan van der Woude
Mark Van Doren
Peter van Galen
Dik van Gent
Wim Van Hul
Jessica van Setten
Douwe van Sinderen
Pieter Van Vlierberghe
Folkert van Werven
Ales Vancura
Barbara Vanderhyden
Cari Vanderpool
Alessandro Vannini
Vinay Varadan
Hervé Vaucheret
Elizabeth Veal
Christopher Vecsey
Jan-Willem Veening
Digna Velez Edwards
Marie-Hélène Verlhac
Marc Vermulst
Teva Vernoux
Paul Verslues
Patrik Verstreken
Kevin Verstrepen
David Vetrie
Jean-Philippe Vielle-Calzada
Usha Vijayraghavan
Bjarni Vilhjalmsson
Anne Villeneuve
Neus Visa
Carlo Viscomi
Karen Visick
Franco Vizeacoumar
Adrianna Vlachos
Eszter Vladar
Renate Voit
Steven Vokes
Talila Volk
Sarah Volkman
Albrecht Von Arnim
Jan Willem Voncken
Denis Voronin
Andrea Vortkamp
Damjan Vukcevic
Scott Waddell
Joseph Wade
Paul Wade
Doris Wagner
Simon Wain-Hobson
Ari Waisman
Adam Waite
Aleksandra Walczak
Matthew Waldor
Chris Wallace
Lori Wallrath
Kyle Walsh
Jianmin Wan
Yonghong Wang
Zhao-Wen Wang
P. Jeremy Wang
Liewei Wang
Jue Wang
Zhiyong Wang
Shizhen Wang
Tao Wang
Yuh-Hwa Wang
Lei Wang
Meng Wang
Biao Wang
Xuelu Wang
Hao Wang
Wei Wang
Isabel Wang
Rui Wang-Sattler
Jonathan Warner
Katherine Warpeha
Coral Warr
Jens Waschke
Andrew Waskiewicz
Katja Wassmann
Sebastian Waszak
Yoshinori Watanabe
Yuichiro Watanabe
Chris Waters
Mark Waters
Christopher Waters

Dawn Watkins-Chow
Paula Watnick
Jennifer Watts
Bianca Waud
Joshua Waxman
Scott Weatherbee
Keith Weaver
Caleb Webber
Michael Weedon
Christian Wegener
Michael Wegner
Bin Wei
Stephan Weidinger
Detlef Weigel
Hartmut Weiler
Mylène Weill
Leor Weinberger
Alan Weiner
Ted Weinert
Bruce Weir
David Weisblat
Ronit Weisman
Eric Weiss
Louis Weiss
James Weisshaar
Matthew Weitzman
Ronald Wek
Deneen Wellik
Ralf Wellinger
Frank Wellmer
Michael Welte
Xiaoquan Wen
Adam Wende
Jurgen Wendland
Theodore Wensel
Joel Wertheim
Helena Westerdahl
Pål Westermark
Matthew Weston
Harm-Jan Westra
Judy Wexler
Clinton Whipple
Charles White
Kristin White
Marquitta White
Alexander Whitworth
Carol Wicking
Reed Wickner
Pamela Wiener
Claudia Wiese
Rudolf Wiesner
Andrew Wilde
Julia Wildschutte
Dagmar Wilhelm
Claus Wilke
Thomas Williams
Amy Williams
Peter Williamson
Judith Willis
Andrea Wills
Daniel Wilson
James F. Wilson
Richard Wilson
Ernst Wimmer
Klaus Wimmers
Wade Winkler
Edward Winter
R. Luke Wiseman
Curt Wittenberg
Mariana Wolfner
Sungho Won
Jason Wong
Richard Wood
Will Wood
Philip Woodman
Bas Wouters
Nancy Woychik
Gerard Wright
Gavin Wright
Chao-Ting Wu
Rongling Wu
Yi-Chun Wu
Keqiang Wu
Xiaohua Wu
Chaodong Wu
Mark Wu
Zhihao Wu
Yongrui Wu
Zhe Wu
Gang Wu
Michael Wudick
Anton Wutz
Michael Wyatt
Joanna Wysocka
Youbin Xiang
Xinshu Xiao
Chao Xing
Jin-Rong Xu
Shizhong Xu
Jian Xu
Tongda Xu
Hong-Wei Xue

Chaoyang Xue
Nobuhiro Yamagata
Yoshiharu Yamaichi
Ai Yamamoto
Daisuke Yamamoto
Shinya Yamamoto
Naoki Yamanaka
Kenshi Yamasaki
Jun Yan
Wei Yan
Hua Yan
William Yang
Jian-Rong Yang
Zhaomin Yang
Jing Yang
Lixing Yang
Chengwei Yang
Can Yang
Xiangdong William Yang
Judith Yanowitz
Humphrey Yao
Yin Yao
Yuanqing Ye
De Ye
Bing Ye
Samuel Yeaman
Trevor Yeats
Lamis Yehia
Rui Yi
John Yin
Keiichiro Yogo
Gyeong Mee Yoon
Timothy York
Satoshi Yoshida
Furuta Yoshikazu
Young-Jai You
Hongtao Yu
Jianming Yu
Jenn-Yah Yu
Hong-Guo Yu
Tianwei Yu
Fengwei Yu
Zhengquan Yu
Ling Yuan
Zhenyu Yue
Christopher Yuskaitis
Katherine Yutzey
Donald Zack
Jean-François Zagury
Hani Zaher
Alan Zahler
Noah Zaitlen
Virginia Zakian
Phillip Zamore
David Zappulla
Raz Zarivach
David Zarkower
Daniela Zarnescu
Troy Zars
Ricardo Zayas
Martin Zeidler
Andrew Zelhof
Rolf Zeller
Robert Zeller
Elazar Zelzer
Kai Zeng
Lirong Zeng
William Zerges
Massimo Zeviani
Shuqun Zhang
Baohong Zhang
Xian Sheng Zhang
Yong Zhang
Lin Zhang
Lixin Zhang
Jin-Song Zhang
Dabing Zhang
Yun Zhang
Yan Zhang
Liangran Zhang
Yunde Zhao
Li Zhao
Xiaolan Zhao
Zhong Zhao
Mei Zhen
Deyou Zheng
Bin Zheng
Jie Zheng
Dao-Xiu Zhou
Xiang Zhou
Yun Zhou
Bin Zhou
Qi Zhou
Zhixiong Zhou
Fang Zhu
Nan Zhu
Daniel Zilberman
Stephanie Zimmerman
Robert Zinzen
Huda Zoghbi
Marcella Zollino
Kristen Zorn

Stephan Zuchner
Orsetta Zuffardi
Jian Zuo
